# Melanized focal changes in skeletal muscle in farmed Atlantic salmon after natural infection with *Piscine orthoreovirus *(PRV)

**DOI:** 10.1111/jfd.12995

**Published:** 2019-04-10

**Authors:** Håvard Bjørgen, Randi Haldorsen, Øyvind Oaland, Agnar Kvellestad, Dhamotharan Kannimuthu, Espen Rimstad, Erling Olaf Koppang

**Affiliations:** ^1^ Institute of Basic Science and Aquatic Medicine, Faculty of Veterinary Medicine Norwegian University of Life Sciences Oslo Norway; ^2^ Mowi ASA Bergen Norway; ^3^ Institute of Food Safety and Infection Biology, Faculty of Veterinary Medicine Norwegian University of Life Sciences Oslo Norway

**Keywords:** black spot, inflammation, melanin, melano‐macrophage, red spot

## Abstract

Melanized focal changes in skeletal muscle of farmed Atlantic salmon (*Salmo salar*) are a major quality problem. The aetiology is unknown, but infection with *Piscine orthoreovirus* (PRV) has been associated with the condition. Here, we addressed the pathogenesis of red and melanized focal changes and their association with PRV. First, a population of farmed fish (PRV‐negative prior to sea transfer) was sequentially investigated throughout the seawater period. The fish were autopsied and tested for PRV infection. Muscular changes were described by macroscopy and histology, and a classification system was established. Second, in an experimental infection trial, PRV was injected intramuscularly to induce changes. The farmed fish was gradually infected with PRV. Red focal changes occurred throughout the observation period with a low prevalence regardless of PRV status. Melanized changes were highly diverse and their prevalence increased during the trial. Changes of low macroscopic grade and histological category were more prevalent in PRV‐negative fish. Diffuse granulomatous melanized changes only occurred after PRV infection. No muscular changes were observed in the experimentally challenged fish. Our studies do not indicate that PRV infection causes red focal changes, but seems important in the development of granulomatous melanized changes.

## 1 INTRODUCTION

Melanized focal changes (“black spots”) in skeletal muscle (fillet) are currently the most serious quality problem in Atlantic salmon (*Salmo salar*) farming in Norway, with an estimated presence in about 20% of the fillets at slaughter (Mørkøre et al., 2015). Morphological investigations revealed chronic focal inflammation with melano‐macrophages causing the black/brown colour (Koppang, Haugarvoll, Hordvik, Aune, & Poppe, 2005; Larsen et al., 2012). The changes display the characteristic features of chronic focal necrotizing granulomatous myositis with large extracellular fat vacuoles. These changes were originally attributed to unwarranted vaccine side effects (Koppang et al., 2005), but were later observed in both vaccinated and non‐vaccinated fish (Berg, Yurtseva, Hansen, Lajus, & Fjelldal, 2012; Larsen et al.,^2014^). In all studies on melanized focal changes, samples have been collected at the end of the production cycle, observed as well‐advanced, chronic, melanized changes exposed after filleting.

The industry has suggested a correlation between red (also known as “bleedings”) and melanized focal changes. Compared with the latter, red changes seem rare, but their prevalence is unknown. Presumably, the red changes are located at the same site as the melanized focal changes, which are most frequent in the cranio‐ventral part of the abdominal musculature (Mørkøre et al., 2015). Bjørgen et al. (2015) investigated both red and melanized focal changes at slaughter for presence of PRV using both immunohistochemistry (IHC) and RT‐qPCR. All samples proved PRV‐positive by PCR; however, the virus is ubiquitous in farmed salmon some months after their sea transfer, and as it replicates in erythrocytes (Finstad et al., 2014), the virus can be found in all organs. Immunohistochemistry demonstrated presence of PRV in macrophage‐like cells and in erythrocytes in red focal changes, and in inflamed melanized tissue including even the centre of melanized granulomas. Importantly, transient forms between red and melanized focal changes were also characterized, confirming a link between the two manifestations. Based on their results, Bjørgen et al. (2015) concluded that PRV was a premise for the development of melanized focal changes. Subsequently, these results were challenged by Krasnov, Moghadam, Larsson, Afanasyev, and Mørkøre (2016), suggesting a bacterial component to the pathogenesis, this being based on the detection of transcripts of various prokaryotic rRNA in melanized changes. None of these studies concluded on an initial cause of the condition. The cause of intramuscular bleedings and focal melanization thus remains unknown.

Here, we investigate the sequential development of red and melanized focal muscle changes in a commercial fish population over a period of 15 months. The aim was to reveal the prevalence, severity and anatomical location of red and melanized changes throughout a production period in a seawater farm. Additionally, the melanized changes were systematically classified based on histological differences. We investigated the focal changes for different pathogens with emphasis on PRV, using RT‐qPCR, IHC and *in situ* hybridization (ISH). In addition, we conducted an *in vivo* experiment trying to induce red and melanized focal changes by injecting PRV in Atlantic salmon. Our studies provide novel insight into the development and nature of a serious quality problem in Atlantic salmon production.

## 2 MATERIALS AND METHODS

### 2.1 Field trial

#### 2.1.1 Fish and sampling information

A total of 240 000 Atlantic salmon, Mowi strain, were transferred to sea water at Mowi's location at Svåsand, Hardanger, Norway, in autumn 2015 with an average weight of 110 g. The fish were intraperitoneally vaccinated according to national requirements. Prior to sea transfer, head‐kidney samples from the fish were tested for presence of PRV by RT‐qPCR (*N* = 53) by PatoGen Analyse, Ålesund, Norway. From autumn 2015 to December 2016, seven major samplings were conducted (I–VII): two in 2015 and five in 2016 (Table 1). At least 600 individuals were autopsied at each time point. In order to thoroughly follow the PRV status of the fish, six minor samplings were conducted in addition to the seven main samplings (E1–E6).

**Table 1:** Main samplings I–VII and minor samplings E1–E6

Name/date for sampling	Weeks post‐sea transfer	Number of fish	Average size kg/cm	Selected fish	Sea temperature (°C) (at 0.5 m)	Sea temperature (°C) (at 5 m)	Sea temperature (°C) (at 15 m)
E1 28/09/15	2	59	n.m.	–	12.7	13.7	14.2
I 26/10/15	4	606	0.11/22.1	36	11.0	11.2	11.5
E2 30/11/15	8	60	n.m.	–	6.9	n.a.	n.a.
II 22/12/15	11	620	0.31/30.0	36	7.1	7.7	7.7
E3 19/01/16	15	61	n.m.	–	6.8	6.9	6.9
III 15/02/16	19	608	0.49/35.5	40	3.6	4.8	4.8
E4 15/03/16	23	61	n.m.	–	4..9	6.0	6.0
IV 18/04/16	27	610	0.75/40.8	27	7.3	6.2	6.2
E5 23/05/16	32	60	n.m.	–	12.0	8.3	8.3
V 20/06/16	36	610	1.24/48.7	30	15.3	11.1	11.1
E6 26/07/16	41	63	n.m.	–	18.0	12.0	12.0
VI 13/09/16	48	616	2.9/61.3	33	15.9	15.3	15.3
VII 05/12/16	59	633	4.9/68.7	31	4.9	6.3	6.3

Date, weeks post‐sea transfer, number of fish autopsied, average size and number of selected fish for histology at each time point. Sea temperature at different depths at sampling dates is also included. All types of samples were obtained in I–VII from selected fish, and only blood was sampled in E1–E6. n.m. = not measured.

In all of the main samplings, the collected samples included peripheral blood (in heparin) and gill, spleen and muscle samples (on RNAlater) from the first 60 individuals. These samples were investigated for PRV‐1 by RT‐qPCR. In this work, PRV equals PRV‐1. Between 27 and 40 fish were at each main sampling selected for a closer investigation of macroscopic changes and for histological analyses. Representative numbers of the different macroscopic manifestations present at each sampling were selected for in‐depth qualitative analysis, including between 5 and 8 control fish (fish with no macroscopic abnormalities). From these selected fish, affected muscle and corresponding unaffected muscle were collected on both formalin and RNAlater. Liver, spleen, head kidney, heart and the sideline containing skin and red and white muscle were collected on formalin. In the additional minor samplings, blood from at least 60 randomly selected fish was collected. All RT‐qPCR analyses were performed by PatoGen Analyse.

#### 2.1.2 Macroscopic registrations

**Figure 1:**
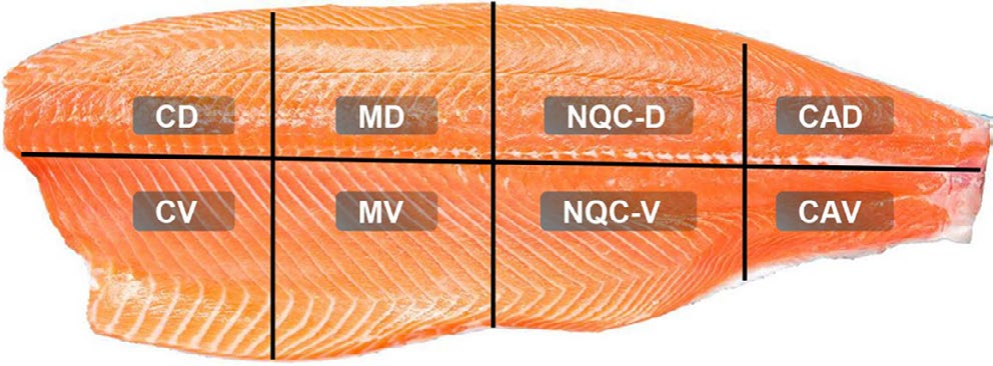
Epaxial and hypaxial muscle (“fillet”) regions referred to by regions in registration of prevalence and description of muscle changes. The fillet is divided into four parts of equal length in the cranio‐caudal direction and further in epaxial and hypaxial parts located dorsal and ventral, respectively, to the lateral line. CD/CV—cranio‐dorsal/cranio‐ventral; MD/MV—mid‐dorsal/mid‐ventral; NQC‐D/V—Norwegian Quality Cut dorsal/ventral; CA‐D/V—caudodorsal/ventral [Colour figure can be viewed at http://wileyonlinelibrary.com]

**Figure 2:**
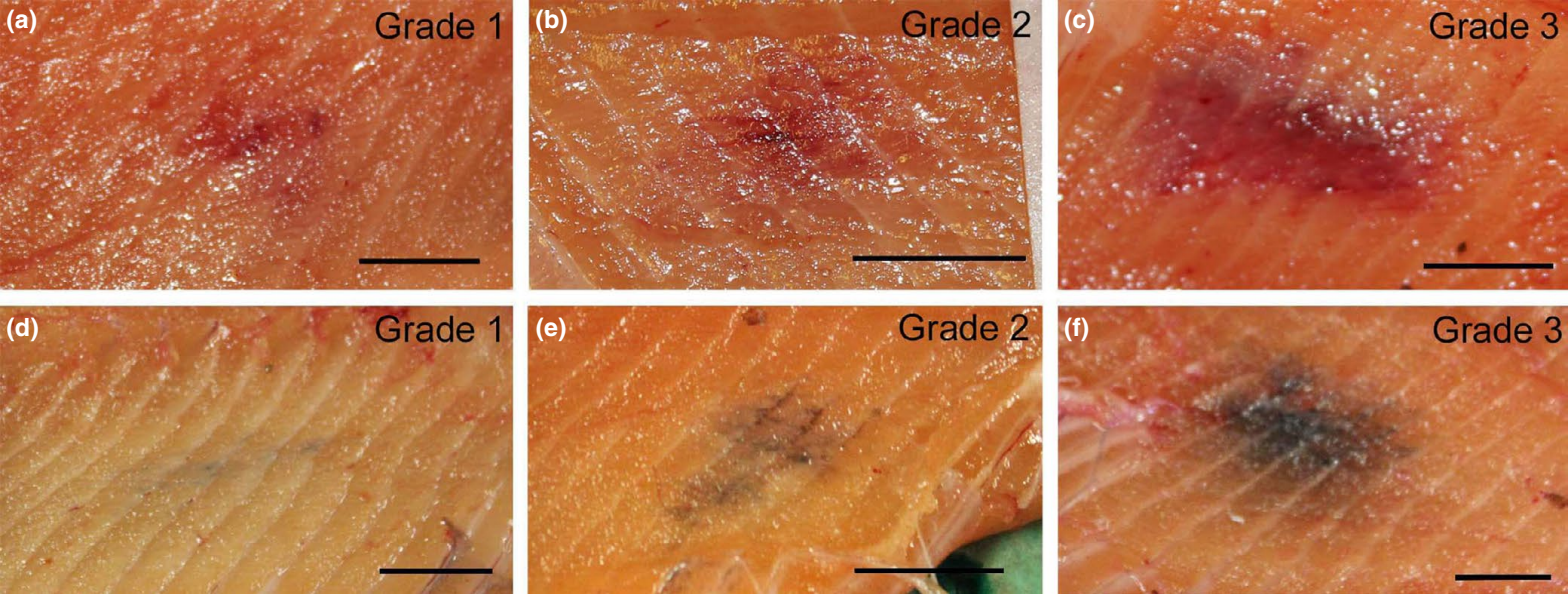
Macroscopic grading of red (a–c) and melanized (d–f) focal changes. Muscle changes were assessed as grade 1, 2 or 3 following the classification system as used by Mowi. Representative pictures from Sampling IV. Grade 1: small in size and/or weak in colour. Grade 2: small, but intense in colour, and/or larger in size. Grade 3: strong colour and large size. Scale = 0.9 cm [Colour figure can be viewed at http://wileyonlinelibrary.com]

After filleting, the changes were registered according to anatomical location (Figure 1), type and grade of change (Figure 2). The grade of both red and melanized changes was assessed according to the scoring system used by Mowi, ranging from grade 1 to grade 3, where 1 was very faint discoloration, 2 was a distinct but not severe discoloration and 3 was a prominent and severe discoloration. Grade 1 would probably not have been registered as a quality abnormality at slaughter by some producers, whereas grades 2 and 3 would have implied a quality reduction of the fillet. Representative examples are given in Figure 2. Statistical calculations on the macroscopic registrations were performed with GraphPad Prism^©^, using Fisher’s exact test to calculate the association between sampling groups.

#### 2.1.3 Histology—standard and special stains

The formalin‐fixed samples were dehydrated through graded ethanol baths, cleared in xylene and embedded in paraffin. Sections were made from all collected samples (all organs) and were subsequent to rehydration and stained with haematoxylin and eosin (HE staining). Three red and melanized changes from each sampling were subjected to Gram, Giemsa and periodic acid–Schiff (PAS) staining. Additionally, five active granulomas (grade 9) were stained for mycobacteria (Ziehl–Neelsen's method). All procedures were performed in accordance with Bancroft and Gamble (2008).

#### 2.1.4 IHC and ISH

Representative changes from each histological category were investigated with IHC for PRV as described previously (Bjørgen et al., 2015), using an antibody targeting the PRV σ1 protein. Briefly, sections were rehydrated and autoclaved. Inhibition was done with phenylhydrazine and blocking with goat normal serum diluted in 5% BSA/TBS (bovine serum albumin/Tris‐buffered saline). The primary antibody was diluted in 1% BSA/TBS (dilution 1:700) and incubated for 30 min at room temperature. The sections were further incubated with an anti‐mouse secondary antibody (Dako EnVision kit) and developed with AEC to evoke colour (red). PBS was used to wash between each step.

To detect viral RNA in the melanized changes, the slides underwent hybridization with RNAscope probe against a portion of PRV‐1 genome segment L1 coding for PRV core shell (Advanced Cell Diagnostics, catalogue #705151). The method was performed using RNAscope (RED) (Advanced Cell Diagnostics, Newark, CA, USA) according to the manufacturer's instructions. The method has previously been used to detect PRV RNA in Atlantic salmon Di Cicco et al., 2018). A heart section from PRV‐negative fish from a previously published challenge study was also included as a negative control (Finstad, 2014). From the same trial, a heart section from a PRV‐positive fish with severe epicarditis was used as a positive control. RNAscope probe against the bacterial gene dapB (#701021) was used as a negative control on duplicates of the same sections to confirm absence of background and/or non‐specific cross‐reactivity of the assay.

### 2.2 Experimental challenge study

A challenge experiment was performed at VESO Vikan aquatic research facility (Vikan, Norway). The Norwegian Food Safety Authority (NFSA), in accordance with the European Union Directive 2010/63/EU, approved the experiment. The experiment included 384 unvaccinated seawater‐adapted Atlantic salmon, SalmoBreed strain. The fish were fed standard commercial food, kept in particle‐filtered and UV‐treated sea water at 12°C and 34 ‰ salinity, anaesthetized by bath immersion in benzocaine chloride (2–5 min, 0.5 g/10 L) prior to handling and killed with an overdose of benzocaine chloride (1 g/5 L). No fish were positive in a PCR screening for selected salmon pathogens prior to the initiation of the experiment.

In Tank 1, Group 1 was injected intraperitoneally (i.p.) with pelleted blood cells containing high loads of PRV (Lund et al., 2016), Group 2 was injected intramuscularly (i.m.) with purified PRV (Lund, 2016), and Groups 3 and 4 were controls given PBS and no treatment, respectively. In Tank 2, Groups 5–7 were given i.p. PRV‐infected blood cells, purified PRV and Renibacterium salmoninarum, respectively, that all had been heat‐inactivated at 85°C for 25 min. In Tank 2, Groups 8 and 9 were controls given PBS and no treatment, respectively. The fish were observed for 18 weeks post‐challenge (wpc), and six fish of each group were sampled at 3‐week intervals. At each sampling, heparinized blood, spleen, kidney, heart and skeletal muscle tissues were collected in RNAlater and in formalin. The fish were filleted and white muscle examined visually for red or melanized changes. RNA was isolated and RT‐qPCR for PRV was performed as described previously (Wessel et al., 2017).

## 3 RESULTS

### 3.1 Field study

#### 3.1.1 PRV detection by RT‐qPCR

**Figure 3:**
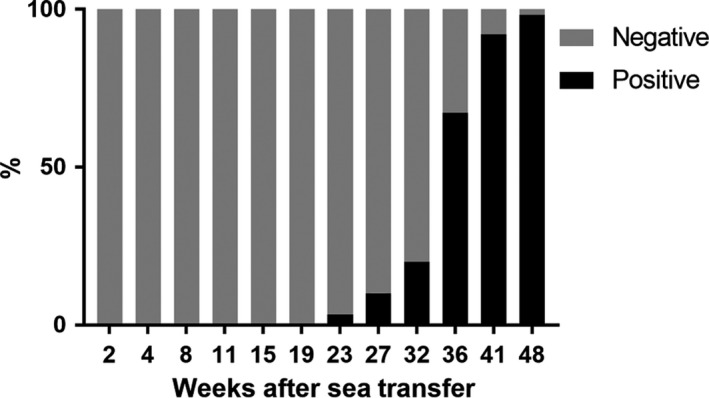
Percentage of PRV‐negative versus PRV‐positive farmed fish tested in peripheral blood by RT‐qPCR. Fifty‐nine to sixty‐three fish were randomly selected in each sampling and analysed for PRV. The bars show that the percentage of PCR‐positive fish increased steadily from detection. The Ct values in PCR‐positive fish varied substantially, from 14.5 up towards cut‐off value. Most Ct values in positive fish were in the range 20–30. Blood samples were not analysed (n.a.) in fish from Sampling VII (59 weeks after sea transfer)

PRV was detected neither in head kidney from 53 fish prior to sea transfer nor in blood from fish from the first three main samplings (I–III) after transfer, that is, during a period of 19 weeks in sea water. Thereafter, the prevalence increased from 10% (6/60 fish) in Sampling IV (Ct values between 14.2 and 35.5), to 67% (41/61 fish) in Sampling V (Ct values between 17.0 and 35.8) to 98% (59/60 fish) in Sampling VI (Ct values between 15.5 and 31.7) (Figure 3).

In addition to blood, at least 30 gill, spleen and muscle samples were analysed from each sampling. PRV was detected in the gills from three fish in Sampling II (Ct values between 36.5 and 36.8), two fish in Sampling III (Ct values between 36.4 and 36.7) and 21 fish in Sampling IV. Spleen and muscle samples remained negative until Sampling IV.

#### 3.1.2 Macroscopic registrations

**Figure 4:**
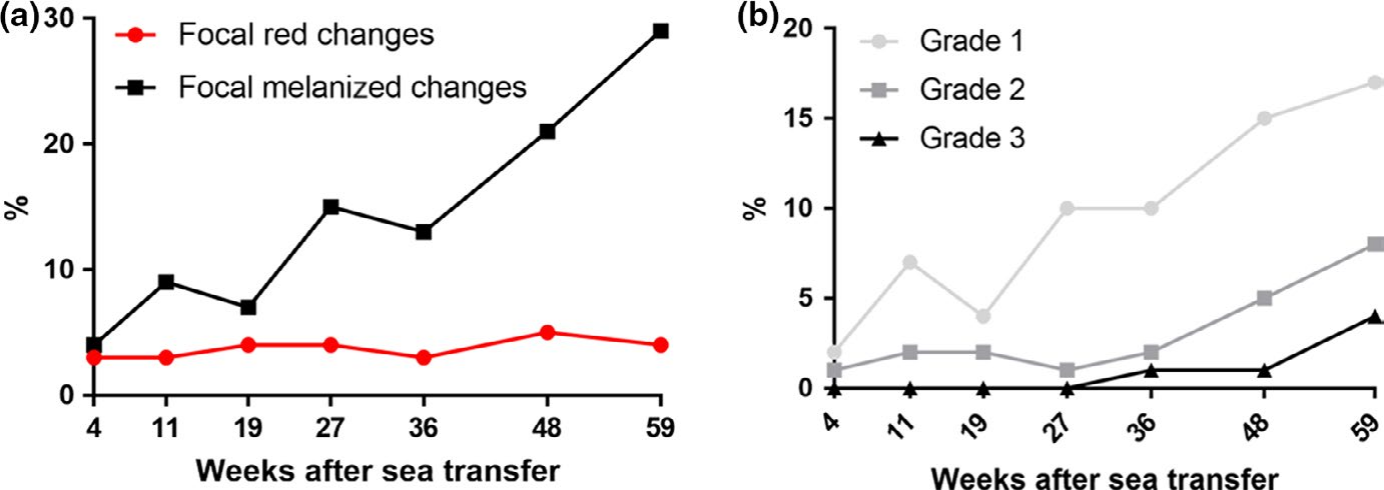
Graphs showing the prevalence of (a) red and melanized focal changes and (b) different melanized macroscopic grades at each sampling shown as weeks after sea transfer. (a) Prevalence of red (red line) and melanized focal changes (black line) in autopsied fish from Samplings I–VII, independent of macroscopic grade. (b) Prevalence of grade 1–3 melanized changes in fish from Samplings I–VII [Colour figure can be viewed at http://wileyonlinelibrary.com]

Both red and melanized focal changes occurred in all samplings (I–VII). Ninety‐two per cent of these changes were in the cranio‐ventral and mid‐ventral regions of the fillet. Red focal changes macroscopically graded 1 to 3 occurred in all samplings. Their prevalence and severity remained stable throughout the observation period with a prevalence of about 4% for each sampling (Figure 4). The melanized focal changes first appeared with low frequency and were macroscopically graded as limited severity (grades 1–2). At later samplings, melanized focal changes became more frequent and their macroscopic severity increased (Figure 4). From Sampling V onwards, melanized focal changes graded 3 were observed (Figure 4 and Table 2).

**Table 2:** Macroscopic grades and histological categories of melanized focal changes present at each sampling

Main samplings	Weeks post‐sea transfer	Macroscopic grades observed	Histological categories observed
I	4	I, II	1, 2, 4, 5
II	11	I, II	1, 2, 5
III	19	I, II	1, 2, 3, 4, 5
IV	27	I, II	4, 5
V	36	I, II, III	1, 2, 5, 6, 8, 9
VI	48	I, II, III	1, 4, 5, 9
VII	59	I, II, III	2, 4, 5, 7, 8, 9

The numbers show which categories and which macroscopic grades were present at each of the main samplings (I–VII).

A statistically significant increase in the prevalence of melanized focal changes was observed from Sampling I to Sampling II (from 6% to 12%). The prevalence was lower in Sampling III (11%) (not statistically significant, *p* > 0.05).

### 3.2 Histological examination

#### 3.2.1 Red focal changes

**Figure 5:**
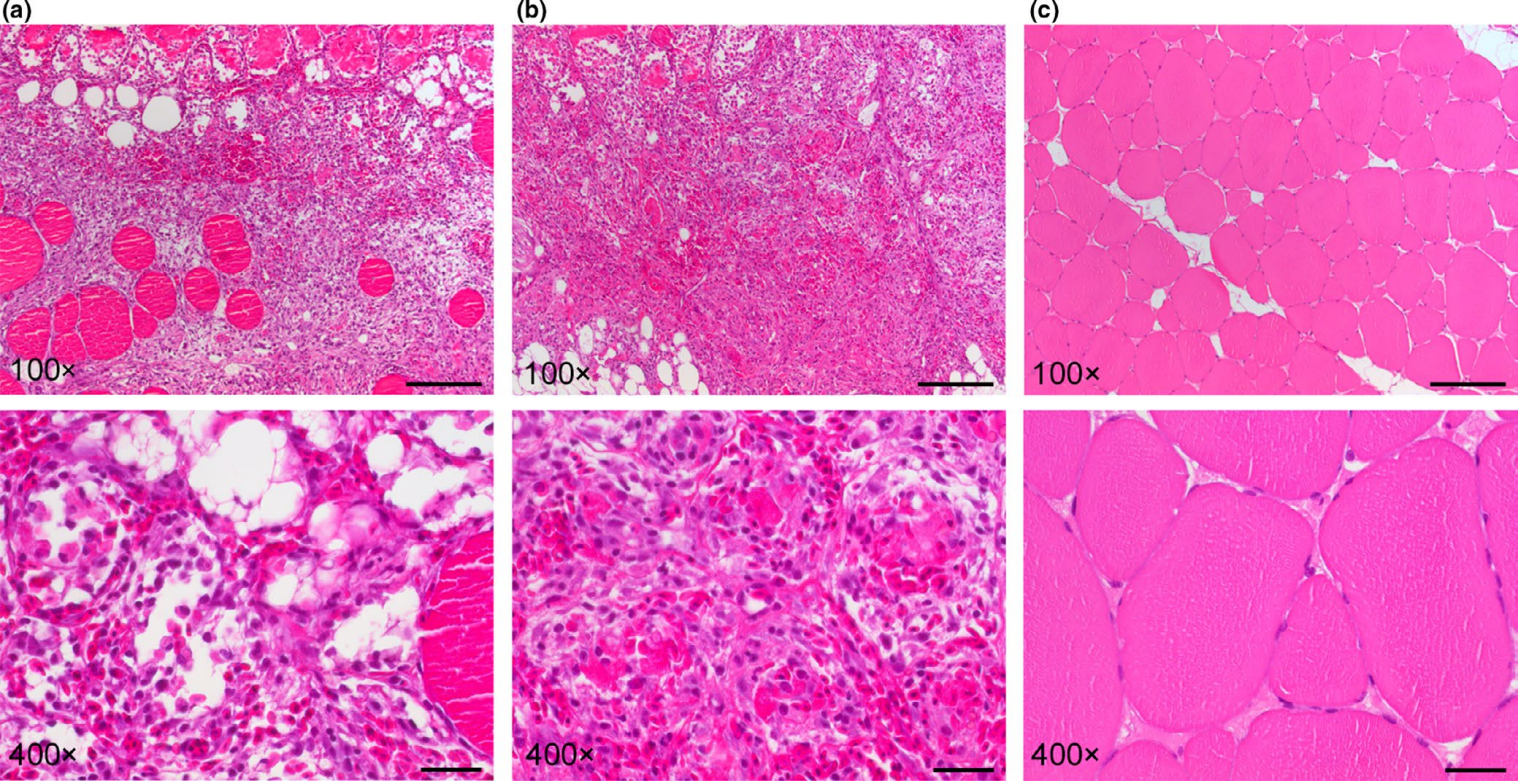
Histopathological characteristics of red focal changes (100X and 400X of the same image). (a) Myocyte necrosis with endo‐ and perimysial haemorrhage. Scattered intact myocytes and vacuoles are present. The 400X image shows endomysial haemorrhage and vacuoles (negative imprint of lipids). Inflammatory cells and activated fibroblasts are present. (b) Severe bleeding and infiltration of inflammatory cells. There is total loss of tissue architecture. Vacuoles of varying size are present in the lower corners. 400X shows endomysial haemorrhage and activated fibroblasts. (c) Control muscle from the same anatomical location shows intact myocytes of various sizes. Adipocytes are limited to the myosepta and occasionally to the endomysium [Colour figure can be viewed at http://wileyonlinelibrary.com]

Red focal changes from all samplings and with macroscopic grades 1, 2 and 3 displayed tissue changes with some variation, which seemed unrelated to the macroscopic appearance, as all variations described here were in all macroscopic grades. The histological findings varied from severe muscle necrosis with sparse haemorrhage to severe bleedings and severe necrosis. Haemorrhage was both endo‐ and perimysial in all changes. Some changes had scattered intact myocytes among macrophage‐like cells and adipocytes (Figure 5a). Other changes were totally devoid of normal tissue architecture occurring with endomysial haemorrhage, leucocyte infiltrates and activated fibroblasts (Figure 5b). Control muscle from the same anatomical location was devoid of any visible pathological changes (Figure 5c).

#### 3.2.2 Melanized focal changes

**Figure 6:**
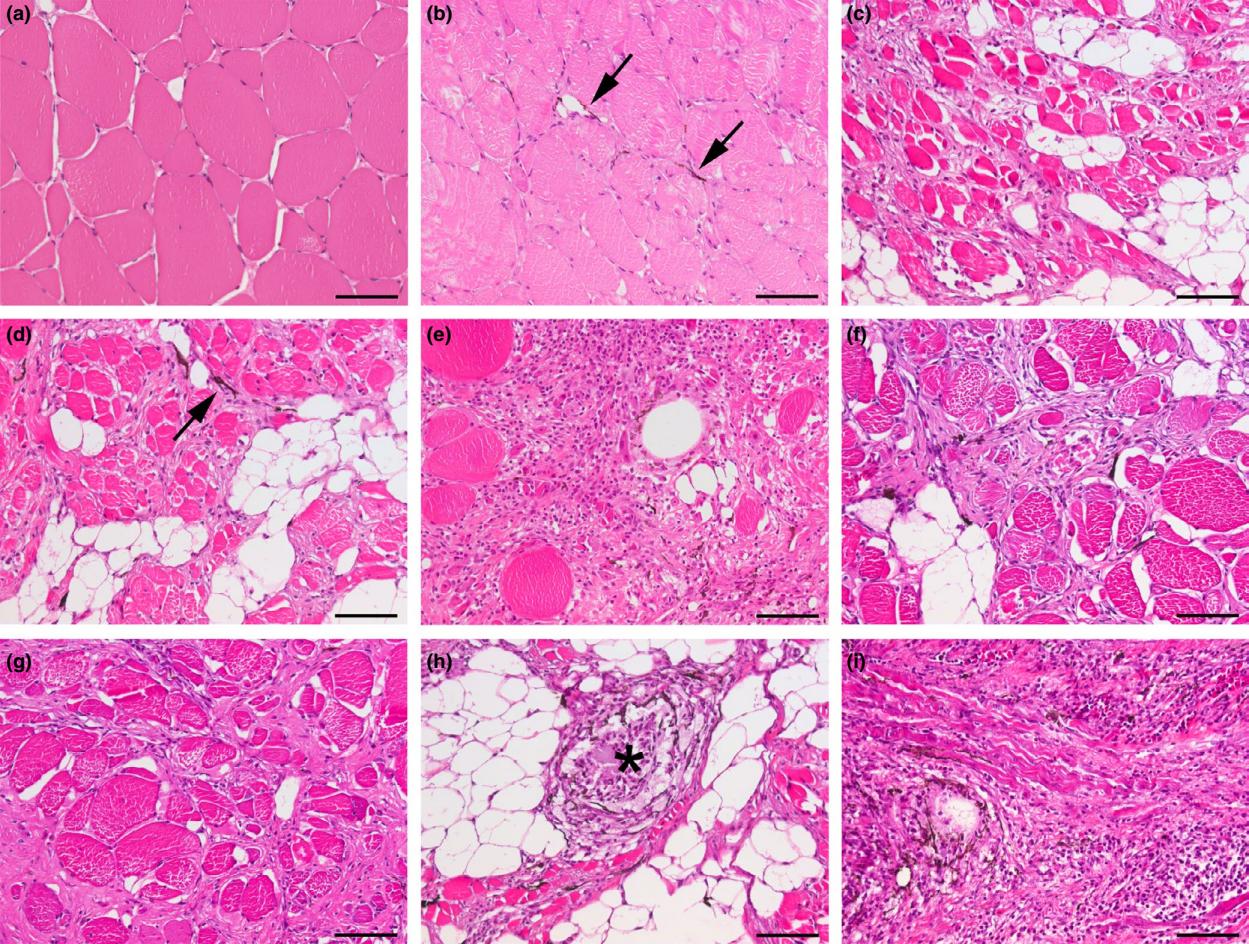
Examples illustrating the nine different histological categories used to classify melanized focal changes. (a) Category 1: no observable changes. Myocytes have a rounded‐to‐hexagonal shape (in cross section), varying sizes and peripheral nuclei. (b) Category 2: interstitial inflammation with endomysial presence of melano‐macrophages that stretch out between (apparently) intact myocytes’ surfaces (arrows). (c) Category 3: fibrosis between (apparently) intact myocytes and adipocytes. No melano‐macrophages are observed. (d) Category 4: area with fibrosis and melano‐macrophages (arrow) between apparently intact myocytes. (e) Category 5: area dominated by fibrosis and inflammatory cells. (f) Category 6: fibrosis and infiltrates of inflammatory cells including melano‐macrophages. (g) Category 7: fibrosis without presence of melano‐macrophages. (h) Category 8: focal granulomatous inflammation. Asterisk indicates the centre of a granuloma. (i) Category 9: diffuse granulomatous inflammation replacing normal tissue in large area. Scale bars = 50 µm [Colour figure can be viewed at http://wileyonlinelibrary.com]

Melanized focal changes were examined and classified into categories from 1 to 9 indicating the possible sequence at which they develop (Figure 6). A set of classification criteria was defined in order to differentiate different pathological features in the melanized changes in HE‐stained sections. As melanized changes are polyphasic and different stages of inflammation might occur in the same section, the changes were categorized according to the dominating feature, in compliance with one of the histological categories. Thus, the macroscopic manifestations were histologically classified into the following categories: (a) no histological changes; (b) melano‐macrophages in the endomysium between apparently intact myocytes; (c) fibrosis in the endomysium without detection of melano‐macrophages; (d) fibrosis in the endomysium with melano‐macrophages; (e) melano‐macrophages, fibrosis and presence of inflammatory cells in the endomysium; (f) (anticipated) old scar tissue with presence of inflammatory cells; (g) old scar tissue with presence of inflammatory cells and melano‐macrophages; (h) focal granulomatous inflammation with presence of melano‐macrophages; and (i) diffuse granulomatous inflammation with myocyte necrosis and myocyte regeneration and presence of melano‐macrophages. The changes previously described by Larsen et al. (2012) would correspond to categories 8 and 9. The other categories have not previously been described.

#### 3.2.3 Macroscopic and histological results combined

When combining the macroscopic manifestations with the histological classification, diffuse granulomatous changes were only encountered in macroscopic manifestation grades 2 and 3 (Table 2). Further, in macroscopic manifestations graded 1, individuals with no observed histological changes were present (Table 2). However, grade 1 macroscopic changes displayed big variation in histological appearance, where all categories of histological changes were detected except diffuse granulomatous inflammation (Table 3). In macroscopic changes graded 2, diffuse granulomatous inflammation (category 9) could be detected; however, most of the category 9 changes were classified as grade 3 changes (Table 3).

**Table 3:** Number of melanized focal changes in each macroscopic grade (1–3) and microscopic category (1–9)

	1	2	3	4	5	6	7	8	9
Grade 1	9	10	1	6	6	1	3	1	0
Grade 2	0	7	0	6	8	1	0	3	7
Grade 3	0	0	0	1	2	1	0	0	5

#### 3.2.4 Other organs

No pathological changes occurred in the liver, spleen or head kidney at any of the samplings. Sections with pancreatic tissue, peritoneum and pyloric caeca revealed a moderate‐to‐severe peritonitis in the first two samplings (I and II) with a decreasing severity in the following samplings and a barely detectable level of severity in the last two samplings. This is in accordance with reactions towards intra‐abdominal vaccination (Mutoloki, 2004). The sideline including red and white muscle revealed scattered inflammatory foci in red muscle in some of the fish in Samplings I and II. Such changes occurred in 11 out of 31 fish in Sampling VII. The white muscle was generally unaffected. Sections of atrium and ventricle from Sampling I were without histological changes. In Sampling II, 3 out of 36 individuals had inflammatory infiltrates in the ventricular myocardium. In Sampling VII, similar changes occurred in 11 out of 31 individuals. The changes could not be associated with the presence of red and melanized changes. Both affected and apparently unaffected hearts occurred among fish with grade 3 changes.

#### 3.2.5 Special stains

Gram, Giemsa, PAS and Ziehl–Neelsen staining did not reveal any microorganisms in red or melanized changes.

#### 3.2.6 IHC and ISH

**Figure 7:**
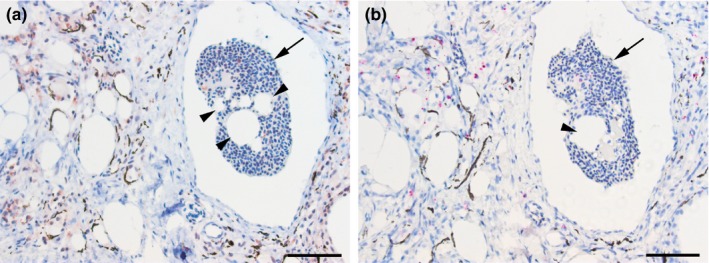
PRV presence in serial sections of a melanized focal change, category 9, with (a) IHC and (b) ISH. (a) PRV‐positive cells (red) are present in the inflamed melanized tissue and in the blood (arrow). Note the adipocytes and vacuoles surrounded by elongated melano‐macrophages. A negative imprint of lipid‐resembling droplets is also evident within the blood in the vessel (arrowheads). (b) PRV‐positive cells (pink) in the same locations as detected PRV by IHC. Staining is evident in the inflamed melanized tissue, but also in the peripheral blood (arrow). A negative imprint of lipid‐resembling droplets is indicated (arrowhead). Scale bars = 50 µm [Colour figure can be viewed at http://wileyonlinelibrary.com]

All granulomatous muscle changes were immunopositive for PRV, in concordance with earlier findings (Bjørgen, 2015), using an antibody targeting PRV protein (Figure 7A). Other, non‐granulomatous, changes could appear both with and without PRV detection. ISH showed positively stained cells in the same locations and a similar distribution pattern as for IHC; however, detection of PRV σ1 proteins was generally more abundant than the detection of PRV RNA (Figure 7B).

### 3.3 Experimental challenge study

The Ct values of PRV RNA in blood cells in the fish injected with PRV, that is, Group 1 injected with PRV‐infected pelleted blood cells and Group 2 injected with purified PRV, were 22–24 at 3 wpc and approximately 30 at all later samplings. A few fish, mainly in Group 2 (injected with purified PRV), became PRV‐negative at 12 wpc. In the cohabitant groups, that is, Groups 3 and 4, there was no detectable virus at 3 wpc, but from 6 wpc and thereafter, all fish in both groups had a Ct of approximately 20 in all samples, with a slight decreasing trend with time. All fish were positive in these groups after 3 wpc. The fish in the cohabitant groups, that is, those infected by natural route, had higher virus loads than the groups where the virus had been injected. Furthermore, there were no indications of clearing of the virus infection during the experiment for the cohabitant groups. In the groups of Tank 2, that is, Groups 5–9, where no infective material was administered, there was no detectable virus throughout the experiment.

No melanized focal changes occurred in any of the groups at any sampling after filleting; that is, melanized changes were not induced by experimental infection of PRV either in purified form or as in erythrocytes or as heat‐inactivated PRV or heat‐inactivated bacteria given i.m.

## 4 DISCUSSION

This study describes the time‐course development of melanized focal changes in a population of farmed Atlantic salmon in a regular commercial field setting during a PRV infection. Investigations on red and melanized focal changes have so far been conducted on material collected at slaughter; hence, the prevalence and severity of such changes during the production cycle in sea water have remained unknown. Here, we provide new information on the development of red and melanized changes from a large‐scale field trial thought representative of most commercial fish farms in Norway. The presence of different features in melanized changes throughout the production cycle led to the establishment of a histological classification system. We also monitored the PRV status of the fish to further investigate the association between PRV infection and melanized focal changes described by Bjørgen et al., 2015. Our results strengthen the hypothesis for a transition from acute red to chronic melanized changes and further support the involvement of PRV in advanced melanized focal changes. *In situ* hybridization studies on PRV showed a great correlation with previous results (Bjørgen et al., 2015) and argue that PRV both persists and replicates in melanized focal changes, possibly explaining their chronic nature. Importantly, we also show that red focal changes and less severe melanized changes may occur without PRV presence.

RT‐qPCR of blood samples became positive for PRV (2/61) in the fourth minor sampling (E4, 23 weeks post‐sea transfer). Thereafter, the prevalence of PRV‐positive blood samples increased steadily, and at the end of the observation period, all tested fish were PRV‐positive. This indicated that PRV was exposed to the fish population after sea transfer and that it took at least 37 weeks from the initial PRV detection in gills (Sampling II) to widespread distribution and viraemia in the majority of the fish (Sampling VI). PRV belongs to the reoviruses; reo is an acronym for respiratory, enteric orphan, reflecting that these viruses were originally detected in the respiratory and enteric organs and that eventual causation of specific diseases was not evident. Enteric uptake of PRV has been indicated earlier (Hauge et al., 2016); however, these authors found no indication of oral entry as the fish were infected only through the intestine after anal intubation. Our finding that PRV was detected in gills before other sites is consistent with the gill as a port of entry for the virus. However, the Ct values from gills were high at the first positive samplings and one should be cautious to determine a port of entry based on that alone. As blood samples were negative in the same samplings, systemic infection with viraemia was probably not yet present. The density of fish farms was high in the area where the study farm is situated and rivers with wild migrating salmon are numerous, giving ample possibilities of spread of PRV through water or through contact with wild or escaped Atlantic salmon (Garseth, Fritsvold, Opheim, Skjerve, & Biering, 2013; Madhun et al., 2018).

The prevalence and the macroscopic and histological appearance of red focal changes remained remarkably stable throughout the observation period regardless of fish size, time post‐vaccination, and ambient temperature or PRV infection status of the fish. The majority of the changes occurred cranio‐ventrally in the fillet, that is, in the same region, and with similar gross appearance as melanized changes. The dominating anatomical localization combined with haemorrhages and myocyte necrosis is intriguing and could indicate local physiological dispositions for the placement. It was not possible to determine whether the changes started because of a haemorrhage following myocyte necrosis or vice versa, as a primary myocyte necrosis followed by haemorrhage. In some of the changes, only myocyte necrosis and haemorrhages occurred. In others, thought to be more advanced, varying amounts of leucocytes occurred. All of these histological forms were observed in all samplings. Histological and transcriptional examination for microorganisms in the changes gave no positive results with the exception of PRV, which appeared in muscle samples about 27 weeks post‐sea transfer, while red focal changes occurred throughout the seawater phase. Consequently, there is no evidence that PRV infection induced the red changes.

The melanized focal changes were classified macro‐ and microscopically, and we provide a histological classification system for melanized changes. Investigations revealed a wide variety in the histological appearances within each macroscopic group. Importantly, the pathological features varied in concordance with the time the population had been in sea water. In the present sequential sampling, changes with the low macroscopic category dominated in the early samplings. Granulomatous inflammatory changes in macroscopic grade 3 occurred only in the last three samplings. The overall progression in severity of the spots with time spent in sea water has not previously been described. Due to the varying microscopic picture, all future studies addressing melanized focal changes should be supported by histological investigations.

Previous studies have reported that unvaccinated farmed fish develop melanized focal changes with the same prevalence at slaughter as vaccinated fish (Berg et al., 2012; Bjørgen et al.,^2015^; Larsen et al., 2014). However, in our current study, we observed the prevalence at different time points. In Sampling II, the overall prevalence of melanized focal changes more than doubled from Sampling I. The reason behind part of the increase might be attributed to vaccination and peritonitis (Mutoloki et al., 2004). Vaccine‐induced side effects (moderate peritonitis) were detected in all fish from Samplings I and II and showed a decreasing severity in the following samplings. Peritoneal melanization is common in vaccine‐induced peritonitis (Poppe & Breck 1997), and one could speculate that this temporary melanization of the peritoneal wall could be registered as a low‐grade melanized change. This fits well with the fact that no severe melanized focal changes (grade 3) were observed at these samplings and that some muscle samples graded 1 showed no histological changes, perhaps indicating only melanization in the peritoneum. We thus believe that vaccination may account for the increased prevalence in Sampling II and that these changes may be of a temporary character without affecting the final prevalence at slaughter.

In some cases, we only encountered melano‐macrophages that dispersed among seemingly unaffected myocytes. Their presence most likely explains the observed macroscopic discoloration. Other inflammatory changes were not observed. One may think that such manifestations may indicate the last phases of haematoma clearance. PRV was not detected in all red focal changes and, therefore, a plausible hypothesis is that in the absence of PRV, the healing process with invading macrophages will clear the changes. Conversely, PRV infection of leucocytes and possibly other cells at the site impairs the clearing process. The virus not only persists but also replicates in cells in the changes and the inflammatory process becomes chronic, as seen by the combined results of ISH and IHC. The lack of ability to eliminate the infection induces formation of granulomas attempting to seal off intruding antigen. This is supported by the ISH findings of replicating virus in all granulomatous changes examined. The melanin would protect the surroundings from the oxidative effect caused by the continuous inflammatory responses in the granulomas; that is, the more melanin and darker the spots, the more severe histopathological changes, which was supported by our findings. It would be ideal to compare these results with a population of fish remaining PRV‐negative throughout the seawater period, but due to the widespread presence of PRV in Norwegian fish farms, such material remains unidentified. Though our results show that PRV is persistently present and replicates in macrophage‐like cells within the chronic granulomatous changes, an alternative hypothesis would be that PRV in such changes is coincidental. The presence of PRV in granulomatous changes could be due to the widespread PRV‐infected cells in the fish and not necessarily correlate with causation. This would imply another aetiology, and given the inflammatory response and granulomatous changes observed, this cause would most likely be of infectious origin. However, screening for viruses and bacteria has so far found that PRV is the only infectious agent consistently present in granulomatous melanized changes.

In the current study, 92% of all melanized changes were located in the cranio‐ventral and mid‐ventral parts of the fillet. It has to be speculated why the majority of changes occur at this site and whether the initial cause might originate in certain anatomical differences throughout the musculature. Interestingly, the percentage of fat content is considerably higher in these locations (47%) as compared to the more dorsal parts of the fillet (9%–18%) (Einen & Skrede, 1998). We did detect a negative imprint of extracellular lipid‐like accumulations in both red and melanized focal changes and even in the lumen of blood vessels (Figure 7). The content of fat in the diet fed to farmed salmon has increased dramatically over the years, from about 15% in the 1980s to 35% today (Tacon, 2005). Concurrently, the amount of marine ingredients has dropped from around 90% in 1990 to 13% in 2013 (Ytrestøyl et al., 2015). The industry reports that the frequency of melanized changes has increased, partly documented in national surveys (Mørkøre et al., 2015). Another important factor reported to have increased over the same period is the occurrence of pancreas disease (PD) which is known to cause changes in white muscle (Mørkøre et al., 2011); however, melanized changes are present with approximately the same prevalence in the northern part of the country where PD has never been diagnosed. The increase in n‐6 fatty acids from vegetable oils has caused concerns, and it is well known from humans that a high proportion of n‐6 to n‐3 fat in the diet shifts the physiological state in the tissues towards the pathogenesis of many diseases: prothrombotic, proinflammatory and proconstrictive (Simopoulos, 2008). In our study, the frequency of inflammatory changes in red and white muscle and myocardium increased with time. This tendency can possibly be associated with the ongoing PRV infection, but also with the growth of the fish and increased content of fat. An important observation from our previous investigation was that a PRV‐positive population of fish farmed in in‐house tanks was virtually free of both red and melanized changes (Groups F and G in Bjørgen et al., 2015). These fish were considerably smaller than the farmed fish in the other groups as they had a slower growth rate and this may have influenced the storage and distribution of fat in the fillet. Based on these observations, we speculate that the role of both fat in the diet and its distribution and content in fish should be given attention in future studies on melanized muscle changes.

We tried to establish an experimental in‐house model for the induction of melanized focal changes by infecting fish with PRV. Artificial haemorrhages combined with PRV were induced by intramuscular injection of blood immediately after it was drawn from the same individual. The injection was in the dorsal muscles and not the cranio‐ventral muscles and due to practical reasons; the duration of the experiment was 18 weeks, which is much shorter than the production period in sea water. A model for induction of melanized changes would be beneficial for many reasons, but the anticipated multifactorial nature of the development of the melanized changes, including anatomical placement, PRV infection, haemorrhages, myocyte necrosis, duration, feeding procedures and ingredients, should all be considered included (Lund et al., 2018).

The present study has shown that the occurrence of red focal changes in the seawater period seems stable and may turn into melanized focal changes, which accumulate over time. Red and melanized focal changes may be present without PRV being detected, but severe melanized forms were only found in association with PRV infection. The results support that the melanized focal changes in the abdominal region predominantly develop as a consequence of proceeding red focal changes. As these seemingly may occur without PRV, future research should reveal the cause for these changes, and subsequently prevent such changes from developing into chronicity. The prevention of PRV presence and replication seems to be crucial in the latter respect.

## CONFLICT OF INTEREST

The authors declare no conflicts of interest.

## AUTHORS’ CONTRIBUTION

HB planned the study, sampled material, carried out histological investigations (IHC and ISH) and wrote the manuscript; RH and AK sampled field material and commented on the manuscript; ØO planned the study and commented on the manuscript; DK carried out histological investigations (ISH) and commented on the manuscript; ER planned the experimental study, sampled experimental material and commented on the manuscript; and EOK supervised the study, sampled field material, analysed the results and edited the manuscript.
